# Parathyroid surgery during the COVID-19 pandemic: Time to think about the “New Normal”

**DOI:** 10.6061/clinics/2020/e2218

**Published:** 2020-08-14

**Authors:** Fábio Luiz de Menezes Montenegro, Marília D’Elboux Guimarães Brescia, Sergio Samir Arap, Marco Aurélio Valmondes Kulcsar, Marcos Roberto Tavares, Luiz Paulo Kowalski

**Affiliations:** Divisao de Cirurgia de Cabeca e Pescoco, LIM-28, Laboratorio de Cirurgia de Cabeca e Pescoco, Hospital das Clinicas HCFMUSP, Faculdade de Medicina, Universidade de Sao Paulo, Sao Paulo, SP, BR.

Since the first case in China at the end of 2019, the global spread of coronavirus disease (COVID-19) has been rapid, leading the World Health Organization to declare it a pandemic in March 2020 (1). Infected individuals have mild disease in almost 80% of cases. In contrast, 15% of patients with COVID-19 have moderate symptoms, requiring in-hospital care, and 5% have very severe disease, requiring hospitalization in the intensive care unit (2). Although the number of severe cases is relatively low, the spread of severe acute respiratory syndrome coronavirus 2 (SARS-CoV-2) has been exponential, and the unexpected situation has caused the medical resources in many countries to be exhausted, leading to several non-COVID-19-related deaths (3).

It has become clear that, in addition to measures to slow the spread of the disease, health facilities should optimize their use to face this massive challenge (4). The concern of infection of surgeons permeated some medical specialties more than others, with a high risk of infection among head and neck surgeons and otolaryngologists (5). In addition, initial reports of fatal outcomes after surgical procedures in previously asymptomatic patients frightened surgeons (6).

The shortage of treatment resources (including hospital beds, ventilators, health professionals, and personal protective equipment) and the high risk of unfavorable postoperative outcomes in infected subjects led many medical societies to recommend postponing non-emergency operations (7-9).

## International recommendations for parathyroid surgery during the initial response to COVID-19

The Society of Surgical Oncology issued a statement with some recommendations for endocrine surgery on March 30, 2020 (10). This statement endorsed the opinion of the American Association of Endocrine Surgeons for parathyroid surgery. Parathyroidectomy was recommended only for life-threatening hypercalcemia that could not be controlled by medical treatment (11).

## The Head and Neck Service and institutional pandemic preparedness and response planning

At the end of March 2020, the São Paulo State Governor ordered that the Central Institute of the Hospital das Clinicas of the University of São Paulo Medical School be dedicated to only patients with suspected or confirmed COVID-19. The Central Institute has 900 beds, and 300 were prepared for intensive care. The Central Institute is one of the eight specialized institutes of the Hospital das Clinicas complex, with 2,400 beds in total (12).

All elective procedures at the Hospital das Clinicas would be postponed, except those considered time-sensitive or medical emergencies. Thus, patients usually treated at the Central Institute, such as those with hyperparathyroidism (HPT), would be transferred and the operations would be performed at other institutes. At the time, we determined that we should operate upon patients with a total calcium level of 14 mg/dL or higher. In addition, patients with neurological symptoms, cardiac complications, pancreatitis, calciphylaxis, or acute kidney injury would receive urgent treatment, even if they had lower levels of blood calcium. Bone pain would be preferably treated with analgesics (13).

## Parathyroid operations at the institution before the COVID-19 pandemic

**Figure 1:**
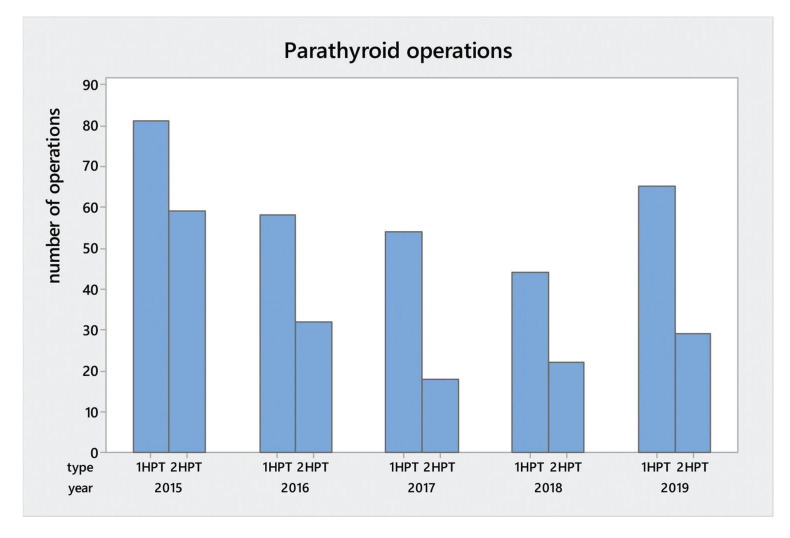
Annual Parathyroid operations before the 2020 COVID-19 pandemic (1HPT=primary hyperparathyroidism, 2HPT=renal hyperparathyroidism).

At the Hospital das Clinicas, parathyroid surgery is performed by a specialized unit of the Head and Neck Division of the Department of Surgery. Most operations are performed by residents under the direct supervision of specialized attending surgeons. In Brazil, Hospital das Clinicas is one of the major parathyroid training and research centers (14). The hospital belongs to University of São Paulo Medical School, a public institution funded by the State of São Paulo. Its main revenue comes from state taxes. Figure 1 shows the number of parathyroid operations from 2015 to 2019 by type and year. The number of total cases decreased from 140 in 2015 to a nadir of 62 in 2018 because of cost restrictions at the institution. The Brazilian financial crises impacted the number of operations (the Gross Domestic Product of the State of São Paulo retracted 4.1% in 2015) (15). The São Paulo State economic activity had been slowly improving, and we had recuperated in 2019, with 94 cases. In 2020, as of March 23, we had 22 patients with primary HPT (18 with adenomas, two with type 1 multiple endocrine neoplasia-related HPT, and two who required reoperations of parathyroid carcinoma) and eight with renal HPT. Following this trend, we would have reached about 120 cases by the end of 2020. However, the pandemic deeply impacted this trend. From March 30 to July 1, 2020, only one patient was operated upon at our institution. She had severe hypercalcemia (total calcium: 18.0 mg/dL, normal: 8.6 to 10.2 mg/dL) and acute renal failure. The operation was successful, with a final pathologic report citing atypical adenoma. The patient’s renal function has improved since the operation, but some degree of permanent loss is expected.

## Mitigation phase and ethical dilemmas associated with parathyroid surgery

When the disease outpaces the containment phase, authorities try to mitigate the problem. The question is when a return to normal activities is possible (16).

Until an effective therapy or vaccine is available, the disease is only being mitigated, and it is impossible to resume “normal” activities. However, some strategies, such as social distancing, large-scale testing, and the use of face masks, may help lower infection rates and balance hospital capacity. Thus, establishing a “new normal” may be necessary to avoid suffering, death, or disabilities due to treatment delays, not resulting from SARS-CoV-2 but caused by the effects of COVID-19 on the healthcare system.

Fortunately, parathyroid carcinoma is rare, and its clinical picture is associated with marked hypercalcemia (17,18). Thus, patients with parathyroid carcinoma will be included in our above-mentioned, initial proposal for parathyroid surgery during the COVID-19 pandemic. However, some patients may have recurrent disease and currently present with lower levels of calcium. Despite that, they have a time-sensitive disease and need to be prioritized.

In contrast, patients with sporadic primary HPT have lower levels of calcium, and several of these patients are asymptomatic. As the disease is usually benign, these patients are typically not prioritized in a situation of limited resources. This line of thought may lead to neglect of specific patients whose calcium levels are near 12 mg/dL and who may have bone pain, nocturia, muscular weakness, or brown tumors. These patients cannot wait indefinitely until the healthcare system returns to “normal.” Thus, we propose that the strict criteria adopted initially be critically reviewed. The same concepts may be applied to patients with type-1 multiple endocrine neoplasia-related hyperparathyroidism. These patients often have mild elevation of serum calcium levels (19). However, they may present with early-onset disease with target-organ damage (20). Of note, these patients are frequently young. Of the patients, 4.3% are younger than 20 years (21). In addition, they may have other tumors requiring surgical treatment or that are better managed after parathyroidectomy, such as gastrinomas (22).

Our initial concern with regard to infected individuals having an unfavorable postoperative course may partly be minimized by preoperative SARS-CoV-2 testing. (23). In some cases, chest tomography may be advisable to detect suspected signs of COVID-19 before the operation. Despite these measures, it is mandatory that the surgeon discusses the risk of acquiring COVID-19 during hospital admission or postoperatively with the patient (24).

## Parathyroidectomy and survival: the case against postponing the surgical treatment of secondary HPT

Unfortunately, many patients with secondary hyperparathyroidism under dialysis are symptomatic. They have a reduced quality of life and usually show significant improvement after parathyroidectomy (25). Although pain relief is immediate postoperatively, pain can be managed safely with medicines until COVID-19 is controlled and a more secure environment is available for these high-risk individuals, as previous chronic kidney disease is a significant risk factor for severe forms of COVID-19 and for death (26).

Brown tumors may pose a challenge in advanced secondary hyperparathyroidism. These benign bone reactions may progress rapidly, usually causing face disabilities. In the hard palate, these tumors often ulcerate and may cause severe bleeding. In rare cases, they involve vertebral bodies, with the risk of fracture and neurological complications. These patients should be included on the priority list for parathyroidectomy during the mitigation phase of the COVID-19 response.

As in time-sensitive diseases, such as cancer, the major factor for advocating parathyroidectomy in patients with chronic kidney disease is indeed survival. Surgeons not involved in the management of secondary HPT are usually unaware of this important fact. Annual death rates may exceed 20% in dialysis patients (27). This mortality rate also applied to young individuals. Dialysis patients aged 24-34 years have the same risk of cardiovascular mortality as do individuals aged 75-84 years in the general population (28). This increased risk of death is mainly due to bone and mineral metabolism abnormalities caused by secondary HPT (29). Parathyroidectomy improves survival. The relative risk of death was lower in patients who underwent parathyroidectomy (in the institution’s experience, it was 0.428; 95% CI, 0.28 to 0.67) (30). Thus, it is important to emphasize the need to also include patients with secondary HPT in the priority list for surgery during the COVID-19 pandemic.

In terms of renal HPT, it is relevant to discuss the problem of persistent HPT in patients with a successful kidney transplant. There is evidence that this problem is not infrequent, affecting up to 62% of transplant patients after one year (31). For them, parathyroidectomy is necessary to protect the kidney graft from hypercalcemia. Even calcium levels below 14 mg/dL may jeopardize the kidney graft in this population. Although a transient rise in creatinine is observed after parathyroidectomy in kidney transplant patients, no significant changes persist in the long term (32,33).

Both patients under dialysis and those with a successful kidney transplant should test negative for SARS-CoV-2 before the operation to minimize postoperative complications. Again, informed consent must clearly address the risk of in-hospital SARS-CoV-2 infection.

In conclusion, the initial proposal for parathyroid operations was adequate and in accordance with the guidelines on early-stage COVID-19 containment by other societies. However, with the passage of time, experience has shown that parathyroid operations should be considered with an expanded view. It is time to think about the “new normal.”
